# Exploring Mechanisms of Multiple Insecticide Resistance in a Population of the Malaria Vector *Anopheles funestus* in Benin

**DOI:** 10.1371/journal.pone.0027760

**Published:** 2011-11-16

**Authors:** Rousseau Djouaka, Helen Irving, Zainab Tukur, Charles S. Wondji

**Affiliations:** 1 International Institute of Tropical Agriculture, Cotonou, Benin; 2 Liverpool School of Tropical Medicine, Liverpool, United Kingdom; 3 Bayero University, Kano, Nigeria; New Mexico State University, United States of America

## Abstract

**Background:**

The insecticide resistance status of the malaria vector *Anopheles funestus* and the underlying resistance mechanisms remain uncharacterised in many parts of Africa, notably in Benin, West Africa. To fill this gap in our knowledge, we assessed the susceptibility status of a population of this species in Pahou, Southern Benin and investigated the potential resistance mechanisms.

**Methodology/Principal Findings:**

WHO bioassays revealed a multiple resistance profile for *An. funestus* in Pahou. This population is highly resistant to DDT with no mortality in females after 1h exposure to 4%DDT. Resistance was observed against the Type I pyrethroid permethrin and the carbamate bendiocarb. A moderate resistance was detected against deltamethrin (type II pyrethroids). A total susceptibility was observed against malathion, an organophosphate. Pre-exposure to PBO did not change the mortality rates for DDT indicating that cytochrome P450s play no role in DDT resistance in Pahou. No L1014F *kdr* mutation was detected but a correlation between haplotypes of two fragments of the Voltage-Gated Sodium Channel gene and resistance was observed suggesting that mutations in other exons may confer the knockdown resistance in this species. Biochemical assays revealed elevated levels of GSTs and cytochrome mono-oxygenases in Pahou. No G119S mutation and no altered acetylcholinesterase gene were detected in the Pahou population. qPCR analysis of five detoxification genes revealed that the *GSTe2* is associated to the DDT resistance in this population with a significantly higher expression in DDT resistant samples. A significant over-expression of *CYP6P9a* and *CYP6P9b* previously associated with pyrethroid resistance was also seen but at a lower fold change than in southern Africa.

**Conclusion:**

The multiple insecticide resistance profile of this *An. funestus* population in Benin shows that more attention should be paid to this important malaria vector for the implementation and management of current and future malaria vector control programs in this country.

## Introduction

Malaria is a major health problem in Benin where it is the main cause of morbidity and mortality particularly among children under five and pregnant women. In this country as across Africa, malaria control relies heavily on vector control through the use of insecticide treated nets (ITNs) and indoor residual spraying (IRS). However, the success of these control methods is threatened by resistance to the main insecticides such as pyrethroids and DDT in malaria vectors. In Benin, resistance to pyrethroids and DDT has been mainly reported in *Anopheles gambiae* and *An. arabiensis* [1], [2], [3] while *An. funestus,* the other major malaria vector, has received very little attention and has been ignored as a vector in the control programs. However, resistance to different classes of insecticides used in public health, such as pyrethroids, carbamates and DDT [4], [5], [6], [7], [8] is increasingly reported in *An. funestus* with fear that this could disrupt control programs against this vector. Indeed, resistance to pyrethroids, DDT and carbamates has been detected in different regions of Africa such as southern Africa (Mozambique) [5], [6], [8], East Africa (Uganda) [7], Central Africa (Cameroon) [9] and West Africa (Ghana) [10]. Little is known about the susceptibility of this vector to insecticides in Benin. Due to the variation of the resistance profiles in *An. funestus* populations across Africa such information cannot be extrapolated from resistance patterns in other countries. For example the resistance pattern observed in Cameroon (DDT and dieldrin resistance) [9] is different to that of southern Africa (pyrethroid and carbamate resistance but full susceptibility to DDT and dieldrin) [5]. It is also different to that of East Africa (pyrethroid and DDT resistance but full susceptibility to carbamates) [7] or to that of Ghana (West Africa) (DDT resistance and moderate pyrethroid resistance) [10].

Record of insecticide resistance observed in *An. funestus* populations so far are mainly caused by metabolic resistance mechanisms either for pyrethroids, carbamates or DDT as neither the L1014F *kdr* mutation nor the G119S *Ace-1* mutation has been detected in this species [6], [7], [10], [11]. Indeed, P450 genes have been found to be associated with pyrethroids resistance [11], [12] and suggested for carbamates as well [4] while mechanisms for the DDT resistance detected in West Africa (Ghana) or East Africa (Uganda) remained uncharacterised. However, the recent detection of the A296S *RDL^r^* mutation in the GABA receptor of *An. funestus* [9] indicates that target site resistance mechanism is also present in this species.

Benin is currently scaling up its malaria control program through Long Lasting Impregnated Nets (LLINs) and IRS. It is crucial that information on susceptibility to main insecticides used in public health and the underlying mechanisms being investigated. This will properly inform control programs of the most suitable insecticides to use and facilitate the design of appropriate resistance management strategies.

In this study, we report the assessment of the susceptibility of one *An. funestus* population from South Benin to several insecticides used in public health and also explore the underlying resistance mechanisms. This information will fill the gap in our knowledge on the resistance distribution in *An. funestus* and help to improve future control programs on this species in Benin.

## Materials and Methods

### Ethics Statement

No specific permits were required for the described study.

### Area of study and mosquito collection

Blood fed An. funestus adult females resting indoor were collected in houses between 06 and 10 AM in Pahou (6° 23′ N, 2° 13′E) in Southern Benin, West Africa. The collection was carried out several times between July 2009 and April 2011. Blood-fed and gravid mosquitoes resting inside houses were collected using aspirators and torches and kept in small cups until fully gravid. The egg-forced laying method recently described [7] was used to induce females to lay eggs. Eggs were sent via courier to the Liverpool School of Tropical Medicine (LSTM) where they were allowed to hatch in small cup with approximately 100ml of water and later transferred to larvae bowls (with 800–1000 ml of mineral water) for rearing. The insectary was kept at 26±2°C with a relative humidity of 80%. The egg batches were pooled and reared together. Larvae were fed abundantly with Tetramin™ baby fish food every day. Water of each larvae bowl was changed every two days to reduce the mortality. The F_1_ adults generated were randomly mixed in cages for subsequent experiments.

#### PCR-species identification

All females used for individual oviposition were morphologically identified as belonging to the *An. funestus* group [13]. A PCR was carried out using the protocol of [14] to confirm that all females that laid eggs were An. funestus s.s.

#### Insecticide susceptibility assays

Insecticide susceptibility assays were carried out using 2-5 day-old F_1_ adults from pooled families following the WHO protocol [15]. Approximately 20-25 mosquitoes per tube with 2-6 replicates were exposed to insecticide-impregnated filter paper for 1h or control paper and then transferred to a clean holding tube supplied with 10% sugar and mortality was determined 24 h post-exposure. We tested the following insecticides: the pyrethroids permethrin (0.75%), and deltamethrin (0.05%); the carbamate bendiocarb (0.01); the organophosphate malathion (5%) and the organochlorines DDT (4%) and dieldrin (4%). The efficacy of the insecticide papers used was verified with the susceptible Kisumu strain of *An. gambiae* to confirm that it provides the expected mortality level.

The effect of the synergist PBO was assessed in combination with 4% DDT due to the high level of resistance observed against this insecticide and also because of previous reports of P450 involvement in DDT resistance [16]. 50 female and 50 male mosquitoes were pre-exposed to 4% PBO paper for 1h and immediately exposed to 4% DDT for 1h. Final mortality was assessed after 24h and compared to the results obtained without PBO.

#### Biochemical assay

Biochemical assays based on the methods described by Penilla et al (1998) [17] were carried out using 25 female adults aged between 1 to 3 days from the Pahou mixed F_1_ mosquito sample. The laboratory fully susceptible An. gambiae Kisumu strain [18] was used as the susceptible control sample since no fresh susceptible strain from An. funestus was available, as done previously [5], [6], [7]. The following enzyme assays were carried out: glutathione-S-transferase (GST), altered acetylcholinesterase (AChE), esterase assays (pNPA rate reaction, α and β esterases) and monooxygenases (P450s). We used a two-sample t-test to compare the results of the biochemical assays between the susceptible strain (Kisumu) and the field samples from Pahou following an adjustment for total protein content. The adjusted mean enzyme level/activity of Pahou was compared to the corresponding Kisumu mean.

#### Sequencing of the Voltage-Gated Sodium Channel (VGSC) gene

Two fragments of the voltage-gated sodium channel (VGSC) gene were amplified and sequenced for permethrin resistant and susceptible mosquitoes from Pahou in order to detect potential mutations associated with pyrethroid/DDT resistance. The first fragment called Ex20 (domain II, segment 6), spans the exon 20 containing the L1014 codon associated with knockdown resistance (kdr) in An. gambiae [19], [20] and the second called Ex27-31 (domain IV, segments 1-5), spans the exons 27 to 31 where the 1534 mutation associated with pyrethroids/DDT resistance has been found in *Aedes aegypti* [21]. Genomic DNA was extracted using the LIVAK method [22] and amplified using the following primers: KdrEx20F GTT CAA TGA AGC CCC TCA AA and KdrEx20R CCG AAA TTT GAC AAA AGC AAA for Ex20, KdrEx27-31F GAA TGC GTT GGT TCA AGC T and KdrEx27-31R TTT GAC GTG CAT GAA AAA TGA for Ex27-31. The primers were designed using the VGSC gene sequence of *An. gambiae*. The PCR was carried out using 10 pmol of each primers and 30 ng of genomic DNA as template in 25 µl reactions containing 1X Kapa Taq buffer, 0.2 mM dNTPs, 1.5 mM MgCl_2_, 1U Kapa Taq (Kapa biosystems). The cycle parameters were: 1 cycle at 95°C for 5 min; 35 cycles of 94°C for 30s, 57°C for 30 s and elongation at 72°C for 1 min; followed by 1 cycle at 72°C for 10 min. Sequences were aligned using ClustalW [23] while haplotypes reconstruction and polymorphism analysis were done using DnaSP v5.10 [24]. The phylogenetic Neighbour-joining trees were constructed using MEGA 4.0 [25].

### Genotyping of field samples for analysis of L1014 *kdr* and G119 *Ace-1* codons

Because direct-sequencing of a high sample size of mosquitoes is expensive a diagnostic method using pyrosequencing was designed to easily genotype the nucleotide position of the known *kdr* (L1014) and *Ace-1* (G119) mutations as done already for *kdr* genotyping in *Culex quinquefasciatus* [26]. Pyrosequencing reactions were carried out according to [27]. Software provided by Pyrosequencing AB was used to design three sequence-specific primers for *Ace-1* and *kdr* mutations (L1014F and L1014S mutations can be detected in the same assay). The sequences to analyze for the genotyping and the dispensation order for both reactions are indicated in Table 1. The lower case of nucleotide “c” and “a” indicates the negative control and should not be incorporated in the target DNA. Target DNA fragments for *kdr* and *Ace-1* were first amplified by PCR using the forward and the biotinylated reverse primers presented in Table 1. The PCR reaction contained forward and biotinylated reverse primers (10 pmol), 1X HotStarTaq buffer, 0.2 mM dNTPs, 1.5 mM MgCl2, 1 U HotStarTaq (Qiagen) and 10 ng genomic DNA. The parameters for amplification were: 1 cycle at 95 °C for 5 min; 50 cycles of 94 °C for 20 s, 57 °C for 30 s and elongation at 72 °C for 20 s; followed by 1 cycle at 72 °C for 5 min.

**Table 1:** *kdr* and *AChE* primer information.

	*Kdr (L1014F* and *L1014S*)	*Ace-1*
Forward primer	TTGTGTTCCGTGTGCTATGC	CCTGTCCGAGGACTGTCTGT
Reverse primer	AAAAACGATCTTGGTCCATGT	ACCACGATCACGTTCTCCTC
Sequencing	TGTAGTGATAGGAAAT	TGTGGATCTTCGGCGG
Sequence to analyse	5′-T C/T A/T GTCGTAAG-3′	5′-C A/G GCTTCTACTCC-3′
Dispensation order for sequencing	5′-TCATcGTCGT-3′	5′-TCAGaCTCT-3′
Product size (bp)	154	165
Allele	C/T//A/T	A/G

Pyrosequencing reactions were performed as described by [27] according to the manufacturer's instructions using the PSQ 96 SNP Reagent Kit (Qiagen) and the sequencing primer shown in Table 1.

### Transcription profiling of candidate detoxification genes

A quantitative PCR (qPCR) analysis of the Pahou population was carried out for two duplicated P450 genes, *CYP6P9a* and *CYP6P9b* previously found to be strongly associated with pyrethroid resistance in the pyrethroid resistant laboratory strain FUMOZ-R [11] and in a field population from Mozambique [6], to assess whether they were also associated with resistance in Benin. The expression profile of the *GSTe2* gene known to confer DDT resistance in *An. gambiae* [16] and *Aedes aegypti* [28] was also analysed. Additionally, *GSTd1-3, a gene* shown as possibly associated with pyrethroid resistance by 454 transcriptome profiling [29] and the P450 gene *CYP6Z1* associated with both pyrethroid and DDT resistance in *An. gambiae* [16] were also tested. The GeXP genetic analysis system from Beckman and Coulter was used according to the protocol in [11]. RNA was extracted using the Picopure RNA isolation kit (Arkturis) from three batches of 10 females resistant to permethrin from Pahou. The same was done for females resistant to DDT and for females not exposed to insecticides (as control for the population) and for 1-3 day-old females from the susceptible laboratory FANG strain (originating from Angola [30] and only available in RNAlater for RNA extraction and therefore not suitable for the biochemical assays). RNA quality was assessed using the Agilent 2100 Bioanalyzer (Agilent technologies). The primers used are listed in Table 2. The expression level of the *RSP7* ribosomal gene (shown to be consistent and with no differential expression between susceptible and resistant [7], [11]) was used to normalise for variation in total cDNA concentration between samples. A two-sample t-test was used to compare the results between samples.

**Table 2:** qPCR primers

Genes	Forward primer	Reverse primer	Expected size (bp)
***CYP6P9a***	AGGTGACACTATAGAATACAATGTGATAAACGAAACACTTCGCAA (common to both genes)	GTACGACTCACTATAGGGACTTTATTATAGATTGGAAGTATCTCA	**490**
***CYP6P9b***		GTACGACTCACTATAGGGACTACAAAAACCCCTTCCGCTGCACC	**504**
***CYP6Z1***	AGGTGACACTATAGAATAGACAATCTAGCGATGGACCATCAGA	GTACGACTCACTATAGGGACGC CTCGGAGAGCGAATCTTC	**430**
***GSTe2***	AGGTGACACTATAGAATACTA CCATCTCTAGCATTATGGGC	GTACGACTCACTATAGGGA GCTTAACCAATTGAATTAATTTC	**233**
***GSTd1-3***	AGGTGACACTATAGAATA CATTCACCGTTGGTACGAGCGC	GTACGACTCACTATAGGGA GCA ACACAAAGAAACACGGAGA	**208**
***RSP7***	AGGTGACACTATAGAATAAGAACCAGCAGACCACCATC	GTACGACTCACTATAGGGAGCTGCAAACTTCGGCTATTC	186

## Results

More than 300 blood fed *An. funestus* females were collected from 2009 to April 2011. Around 160 laid eggs and all were confirmed to be *An. funestus s.s.* by PCR.

### Susceptibility tests

**Figure 1:**
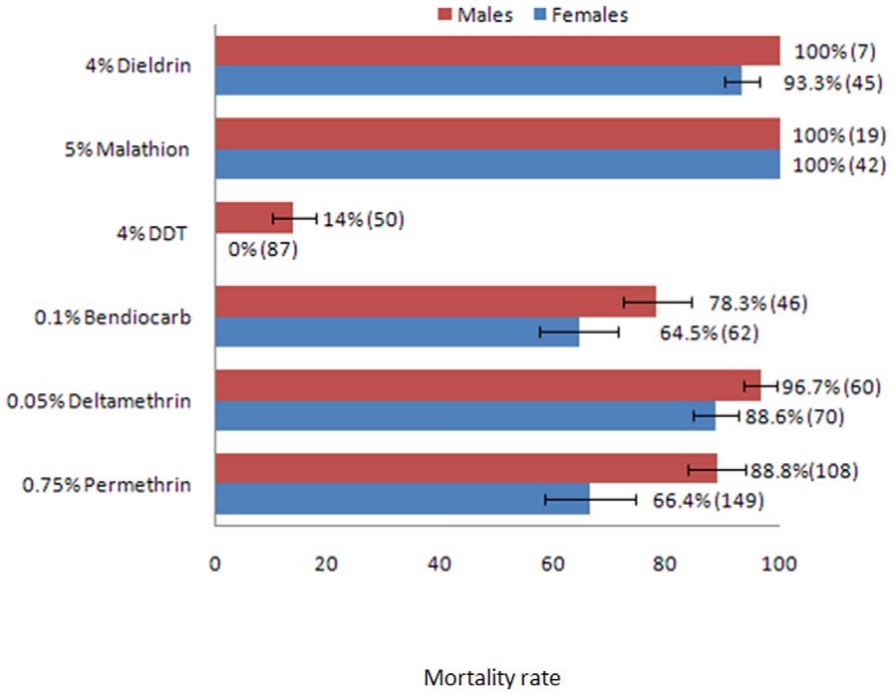
Susceptibility/resistance status of the *An. funestus* population of Pahou to the main insecticides. The numbers in brackets indicate the number of mosquitoes exposed for each insecticide.

WHO bioassays carried out using mixed F_1_ adults revealed that the *An. funestus* population of Pahou was highly resistant to DDT with no mortality observed for females and only 14% mortality for males (Figure 1). The 4% DDT papers used was confirmed to be fully effective with 100% mortality observed with the Kisumu strain of *An. gambiae*.

Resistance was also observed against permethrin (Type I Pyrethroid) for females with a mortality of 66.7% while only a moderate resistance was observed for males with a mortality rate of 88.8%. Exposure to 0.05% deltamethrin, a type II pyrethroid insecticide, indicated that the *An. funestus* population from Pahou is moderately resistant to this insecticide with 88.6% mortality for females and 96.7% for males. This resistance pattern is different from results from East Africa and Southern Africa where higher resistance was always observed for Type II than type I pyrethroids.

Bioassays with 0.1% bendiocarb, a carbamate insecticide, also revealed a resistance against this insecticide with a mortality rate of 65 and 78.1% respectively for females and males.

A very moderate resistance level was observed against 4% dieldrin with 93.3% mortality for females and 100% mortality rate for males although with a low sample size.

A total susceptibility was observed against malathion, an organophosphate, with 100% mortality for both females and males. No mortality was observed in the control tubes.

Due to the high resistance observed against DDT in this *An. funestus* population from Pahou, a synergist assay with PBO was carried out to assess the potential role played by cytochrome P450 genes. Mortality rates for both females and males were very similar to the results observed without pre-exposure to PBO with 4 and 12% respectively for females and males indicating that P450 genes play no or little role in the observed DDT resistance in Pahou.

### Biochemical assay

**Figure 2:**
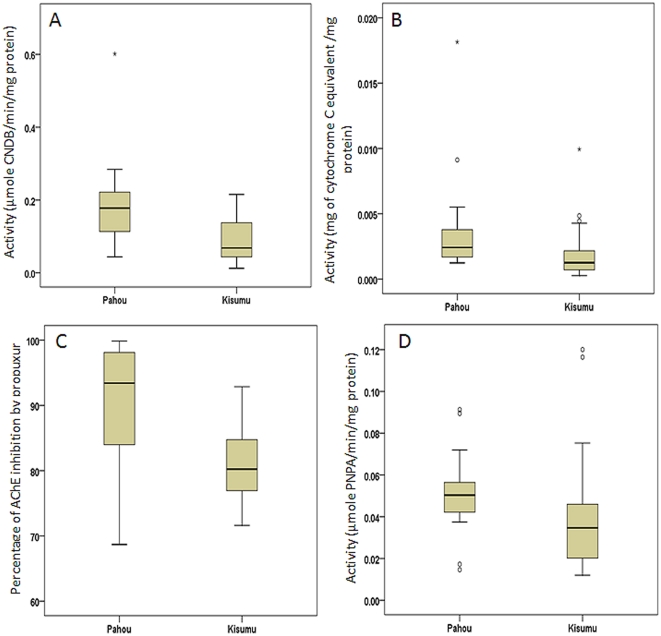
Box plots of results from biochemical assays. The median activity of the An. funestus population of Pahou compared with the An. gambiae Kisumu reference strain is shown by a horizontal bar; the box denotes the upper and lower quartiles. The vertical lines show the full range of the data set; (A) Range of GST activity, (B) Estimated levels of cytochrome P450s (representing monooxygenase activity), (C) Acetylcholinesterase inhibition ranges, (D) Range of esterase activity with the substrate p-nitrophenyl acetate.

A significant increase in the level of both GSTs and cytochrome monooxygenases was observed in the Pahou population compared to the susceptible Kisumu strain. For GSTs, a 2.09-fold increase in the mean quantity of CNDB converted was observed compared to the Kisumu strain (*P<0.0001*) (Figure 2A, Table 3). For Cytochrome monooxygenases, an increase level of 2.53-fold in the amount of cytochrome C equivalents/mg of protein was detected in the Pahou population when compared to the Kisumu strain (*P<0.0063*) (Figure 2B, Table 3).

**Table 3:** Comparisons of average values for a range of biochemical assays between F_1_ adult progeny An. funestus from Pahou population and the An. gambiae Kisumu insecticide-susceptible reference strain.

	MeanPahou	Mean Kisumu	Fold change	*P* value
pNPA	0.051 *[0.014-0.091]*	0.039 *[0.012-0.12]*	1.3	0.05
α-Naphthyl acetate	1.05 10^−05^ *[1.4 10* ^−*6*^ *-6.1 10* ^−*5*^ *]*	1.6 10^−05^ *[2.3 10* ^−*6*^ *-7.3 10* ^−*5*^ *]*		0.18
β-Naphthyl acetate	9.6 10^−05^ *[5.8 10* ^−*8*^ *-2.8 10* ^−*5*^ *]*	6.5 10^−05^ *[2.2 10* ^−*6*^ *-6.9 10* ^−*4*^ *]*		0.03
P450	0.00324 *[1.2 10* ^−*3*^ *-1.8 10* ^−*2*^ *]*	0.0013 *[2.6 10* ^−*4*^ *-4.4 10* ^−*3*^ *]*	2.53	0.0063
GST	0.18 *[0.043-0.6]*	0.086 *[0.012-0.21]*	2.09	0.0001
AChE	90.8 *[68.7-99.8]*	81.2 *[71.6-92.9]*		0.001

A statistically significant difference in the rate of *AChE* inhibition was observed between the two strains (*P<0.001*). But instead of a lower *AChE* inhibition by propuxur rate as expected for resistant samples with altered acetylcholinesterse, a higher rate of inhibition of 90.8 was rather observed in Pahou compared to 81.2 in Kisumu (Figure 2C) indicating that there is probably no altered acetylcholinesterase in the Pahou population.

No significant increase in esterase activity was observed in the Pahou population compared to the Kisumu susceptible strain with either the pNPA (*P = 0.05*) (Figure 2D, Table 3) and the α-Naphthyl acetate substrates (Table 3). However a slightly significant increase (*P = 0.03*) was seen with the β-Naphthyl acetate substrate (Table 3).

### Sequencing of the Voltage-Gated Sodium Channel (VGSC) gene

PCR products of respectively 994bp and 1355bp were successfully amplified and sequenced for Ex20 and Ex27-31 fragments of the VGSC gene in five permethrin resistant and five permethrin susceptible mosquitoes from Pahou. An 837bp and 921bp common sequence was aligned respectively for Ex20 and Ex27-31 for the 10 individuals. The summary of the polymorphism of this fragment is presented in Table 4.

**Table 4:** Summary statistics for polymorphism at the two fragments of the voltage gated sodium channel (VGSC) gene (Ex20 and Ex27-31) in permethrin susceptible and resistant *An. funestus* from Pahou, Benin.

Samples	N	S	Ts	Tv	Single-tons	F	h	π (k)	D (Tajima)	D* (Fu and Li)
**Ex20**										
**susceptible**	10	8	4	4	4	30% (S3, S7)	5 (0,889)	0.0035 (2.93)	-0.34^ns^	-0.59^ns^
**resistant**	10	5	2	3	4	60% (R5)	5 (0,667)	0.0015(1.26)	-1.13^ns^	-1.35^ns^
**Total**	20	10	5	5	6	30% (R5)	10 (0,895)	0.0027 (2.27)	-0.94^ns^	-1.2^ns^
**Ex27-31**										
**susceptible**	10	4	3	1	2	50% (*7)	4 (0,733)	0.0012 (1.1)	-0.82^ns^	-0.34^ns^
**resistant**	10	6	5	1	4	30% (R2)	7 (0,911)	0.0018(1.73)	-0.75^ns^	-0.93^ns^
**Total**	20	8	6	2	6	30% (*7)	9 (0,789)	0.0017(1.63)	-0.93^ns^	-0.92^ns^

N =  number of sequences (2n); S, number of polymorphic sites; F, frequency of the most common haplotype; h, Number of haplotypes (haplotype diversity); π, nucleotide diversity (k =  mean number of nucleotide differences); Tajima's D and Fu and Li's D statistics, ns, not significant.

**Figure 3:**
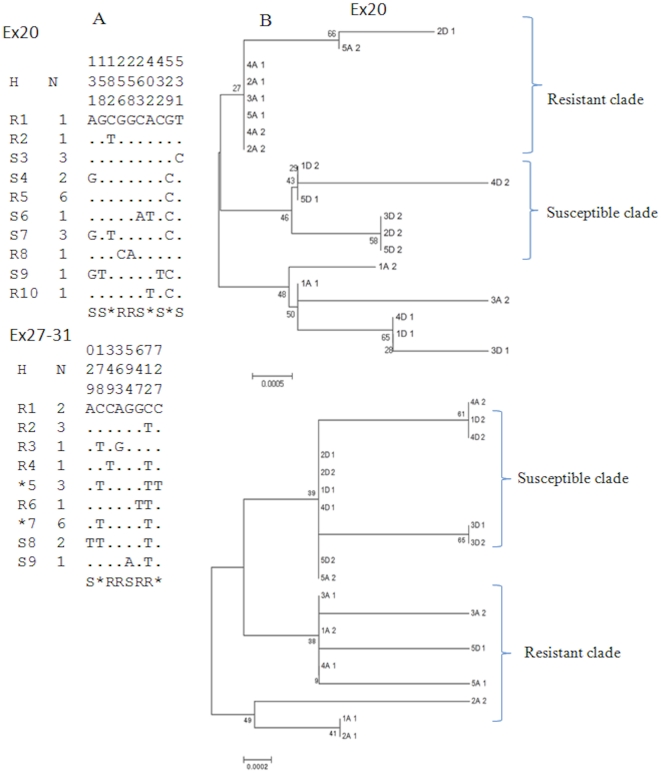
Schematic representation of haplotypes of Ex20 and Ex27-31 fragments (H) of the Voltage-gated sodium channel gene (VGSC) observed in the Pahou population. A) Only polymorphic sites are shown and these are numbered from the beginning of each aligned sequence. Dots mean identity with the first sequence. A number has been given to each haplotype preceded by the letter R or S if it is unique to the resistant or susceptible sample respectively. In case of a shared haplotype, the number is preceded by an asterisk. The column (N) indicates the number of individuals sharing the haplotype. Below the list of haplotypes, R or S indicates the positions that are polymorphic in the resistant or susceptible mosquitoes respectively, while an asterisk marks a position polymorphic in both phenotypes. B) Neighbor-joining tree of the VGSC haplotypes from Pahou showing the clades specific to each phenotype. Haplotypes are named with a number followed either by a letter A for alive (resistant) or D for dead mosquitoes (susceptible), while the last number indicates whether it is the first or second haplotype of the specimen.

Overall 10 and 8 polymorphic sites were observed respectively for Ex20 (5 transitions and 5 transversions) and Ex27-31 (6 transitions and 2 transversions). Slightly less polymorphic sites were observed in resistant than in susceptible samples for Ex20 (5 vs 8) while it was the opposite for Ex27-31 with 4 polymorphic sites in susceptible and 6 in the resistant mosquitoes. No mutation was observed at the 1014 codon often associated with resistance in other insects and overall no polymorphic site was observed in the coding region of Ex20. Two synonymous changes were observed for Ex27-31 but no amino acid change was detected. A similar number of haplotypes was observed for both fragments with respectively 10 and 9 for Ex20 and Ex27-31 (Figure 3A). These haplotype sequences have been submitted to Genbank (Accession numbers: JN248543-JN248562).

The Neighbour-joining phylogenetic tree of each fragment indicated a possible correlation between the haplotypes and the resistance phenotype as haplotypes of resistant individuals were predominant in one clade while the susceptible haplotypes were also predominant in another clade for both Ex20 and Ex27-31. For Ex20, the resistant clade is made of 7 haplotypes from resistant individuals out of a total of 8, while the susceptible clade contains only haplotypes from susceptible individuals (Figure 3B). For Ex27-31, 5 out of 6 haplotypes in the resistant clade belong to resistant mosquitoes, while for the susceptible clade, 9 out of 11 haplotypes come from susceptible individuals (Figure 3B). This correlation suggest that although the 1014 was not identified, that another target-site mutation conferring resistance to permethrin may be located elsewhere in the VGSC gene in the Pahou *An. funestus* population.

A high similarity level was observed between *An. funestus* and *An. gambiae* for Ex27-31 as only 1 amino difference was noticed over 235 amino acids.

### Genotyping of known target-site mutation positions

**Figure 4:**
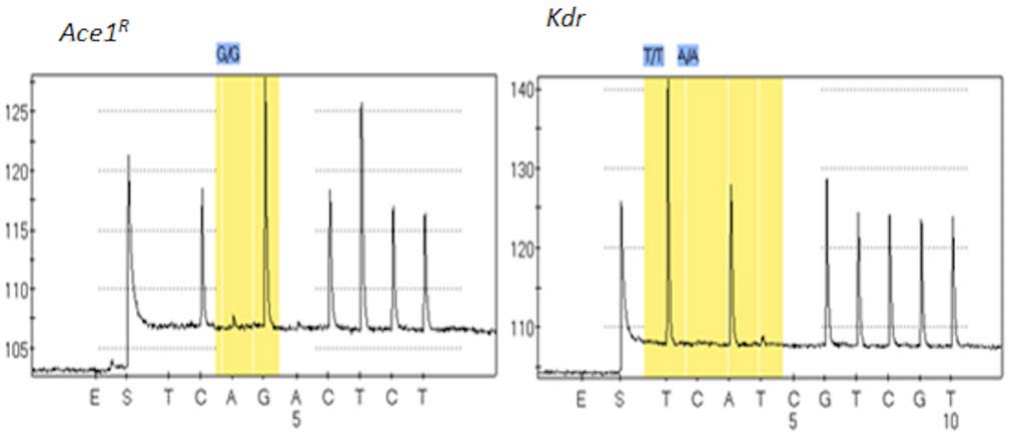
Pyrosequencing pyrogram traces for *AChE* (left) and *kdr* (right) genotyping.

Amplification of the *kdr* and *Ace-1* fragments for the pyrosequencing reaction was successful with bands for *kdr* and *Ace-1* respectively at 154 and 165 bp. Pyrogram traces for fifty field collected mosquitoes detected only the TTA 1014 codon of the voltage-gated sodium channel indicating that they do not have the L1014F (TTA-to-TTT) or L1014S (TTA-to-TCA) *kdr* mutation (Figure 4) commonly found in *An. gambiae*. This confirms the *VGSC* gene sequencing results that the 1014 *kdr* mutation is not present in the *An. funestus* population of Pahou.

The genotyping of the same 50 mosquitoes did not find the G119S mutation with all pyrogram traces showing the GGC codon (Figure 4) which codes for glycine, confirming that the G119S mutation is not present in the Pahou population.

#### Transcription profiling of candidate P450 genes

**Figure 5:**
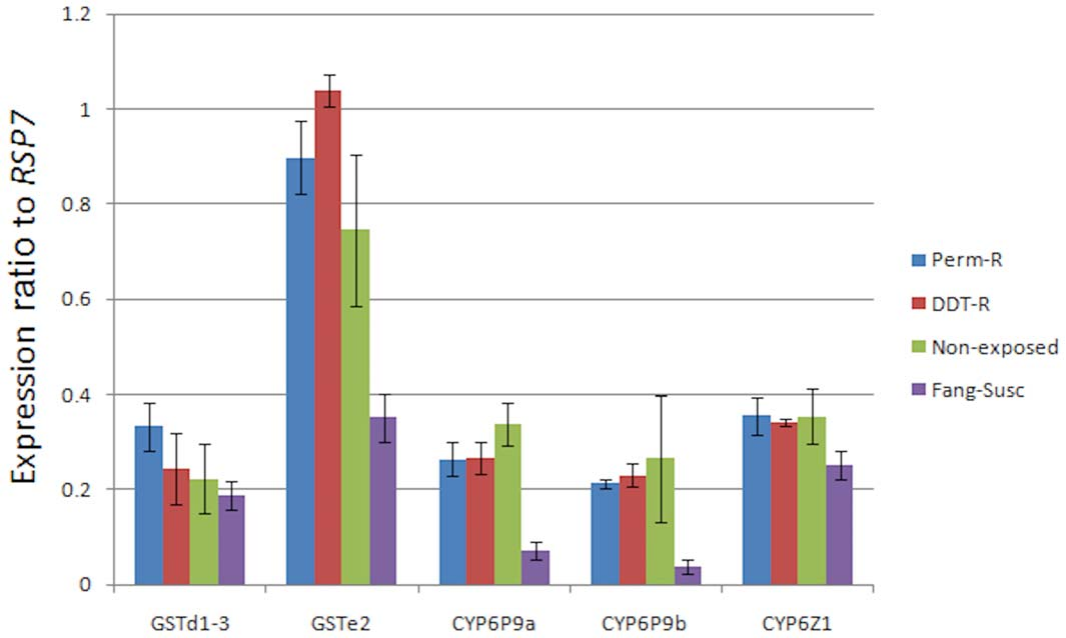
Transcription profile of candidate genes in the Pahou population. Comparison of the patterns of gene expression of five candidate detoxification genes (*CYP6P9a, CYP6P9b, GSTe2, GSTd1-3 and CYP6Z1*) between permethrin resistant (Perm-R), DDT resistant (DDT-R), Pahou mosquitoes not exposed to insecticides (Non-exposed) and the laboratory susceptible strain FANG (FANG-Susc). The normalised expression ratio of each gene against *RSP7* gene is represented on the vertical axis.

Analysis of the expression level of *CYP6P9a* indicated that this gene is significantly over-expressed in the Pahou population compared to the FANG susceptible strain (Figure 5). Indeed, a 3.6, 3.7 and 4.7-fold over-expression were observed when comparing the Pahou samples to FANG (*P<0.01*) respectively for Permethrin resistant, DDT resistant and non-exposed mosquitoes. As *CYP6P9a* has been strongly associated with pyrethroid resistance in southern Africa, the expectation was to observe a higher expression in permethrin resistant samples than in non-exposed mosquitoes. But this was not the case as no significant difference was observed between the three Pahou samples.

A similar pattern was observed for *CYP6P9b* with also a significant over-expression of this gene in the three Pahou samples than in FANG susceptible strain (Figure 5). The level of over-expression is respectively of 5.7, 6.2 and 7.15 fold change for Permethrin resistant, DDT resistant and non-exposed mosquitoes when compared to FANG (*P<0.01*). Again no significant difference is observed between the three samples of Pahou.

*GSTe2* was also significantly over-expressed in the three Pahou samples compared to the FANG susceptible strain with respectively 2.55, 2.95 and 2.12 fold change in permethrin resistant, DDT resistant and Non-exposed mosquitoes (P<0.01). The level of expression of *GSTe2* was significantly higher in DDT resistant samples compared to permethrin resistant and non-exposed mosquitoes (*P<0.01*) while no significant difference was observed between permethrin resistant and non-exposed.

No significant difference was observed for *GSTd1-3* between the Pahou samples and the FANG susceptible strain or between the three Pahou samples.

The difference in expression was moderate for *CYP6Z1* P450 gene between the three Pahou samples and the FANG susceptible strain with respectively a 1.42, 1.37 and 1.41 fold change for permethrin resistant, DDT resistant and Non-exposed mosquitoes. The *P* value associated with this significance was also just below *P<0.05*. No difference was observed between the three Pahou samples.

## Discussion

The DDT resistance level observed in this population (with no mortality recorded in females after 1h exposure) is not only the highest among the insecticides tested in Pahou, but also is the highest level of DDT resistance reported for an *An. funestus* population across Africa until now. No DDT resistance is reported in southern Africa [6] while only a moderate DDT resistance is observed in East Africa [7]. The spread of this DDT resistance to other regions of Africa could reduce the options available for insecticide resistance management of *An. funestus* populations that are already resistant to pyrethroids and carbamates as seen in southern Africa [5]. However, this scenario is unlikely to happen quickly as it has been shown that there is a restriction of gene flow between *An. funestus* populations of West and southern Africa [31], [32]. The high DDT resistance in this West Africa population of *An. funestus* makes it difficult to explain why despite extensive use of DDT resistance has never been observed in southern Africa. We can postulate that this is due to the fact that the molecular mechanism conferring this resistance is completely absent in southern Africa populations and that the restriction in gene flow has not allow it to spread to these populations yet. To confirm this hypothesis, the underlying resistance mechanisms should be fully characterise by identifying the main genes responsible and functionally compare these between the West and southern Africa populations in order to assess differences.

The source of this high DDT resistance in Pahou is unknown but it can be hypothesized that past control programs with DDT may be a likely factor as such resistance is also observed in *An. gambiae* although at a lower level with 37% mortality rate (Djogbenou et al 2010) compared to zero for *An. funestus*. Vegetable farming with use of synthetic pesticides is practiced at Pahou and could implicate agricultural pesticides residues in the selection of this high resistance level. In addition, it is worth mentioning that the locality of Pahou is crossed by the Aheme Lake's streams which sweep and converge several environmental pollutants and pesticide residues from the neighbouring peri-urban cities and farms to the coastal locality of Pahou. Although there is no evidence to date that pyrethroid resistance in adults *An. funestus* results from selective pressure in larvae, it could be different for DDT as this insecticide is more persistent in the environment than pyrethroids. Therefore, it is possible that several ranges of xenobiotics present in these water bodies around Pahou might have contributed also to the selection of this multiple resistance in *An. funestus*However, further investigations are needed to clearly elucidate the main factors contributing to the high levels of resistance recorded in this population of *An. funestus*. It will also be interesting to establish the geographical distribution of this DDT resistance across Benin. DDT resistance in *An. funestus* has been recently reported in Ghana, other West African country, but at a significantly lower level than in Pahou. Indeed, the mortality rate to 4% DDT observed in the *An. funestus* population of Obuasi region in Ghana was around 60 to 80% [10] while no mortality was observed in Pahou.

The present study reports the first case of pyrethroid resistance in An. funestus in Benin. However, this resistance is higher against permethrin (type I pyrethroid) than to deltamethrin (type II pyrethroid). This resistance pattern is different to that observed in southern or East Africa where resistance to type II pyrethroid is higher than to type I [6], [7]. This difference may underline the existence of a different resistance mechanism for pyrethroid resistance in Benin compared to these regions. Moderate permethrin resistance was also detected in another West African *An. funestus* populations from Obuasi in Ghana [10] but no pyrethroid resistance was reported in an *An. funestus* Soumossou in Burkina Faso [33] indicating that this resistance may not yet be widely distributed across West Africa. The level of pyrethroid resistance observed in Pahou is also lower than the level observed in southern Africa notably in Mozambique where only 20% mortality was recorded after exposure of mosquitoes to 0.75% permethrin for 3 hours [6]. Nevertheless, this resistance to pyrethroids is of great concern for malaria control programs with interventions based on LLINs as high level of resistance to this insecticide class is already widespread in *An. gambiae*, the other major malaria vector in Benin [2]. Additionally, there is a risk that if such resistance is not managed properly, it can be further selected by ongoing control interventions such as the pyrethroid impregnated LLINs and IRS to a level that will seriously impact the success of future control programs.

The Pahou populations of *An. funestus* is also resistant to the carbamate bendiocarb but not to the organophosphate malathion indicating that there is no cross-resistance carbamates/organophosphates as seen also in Mozambique [6]. This is further confirmed by the absence of an insensitive acetylcholinesterase (biochemical assay) and the absence of the G119S mutation in the acetylcholinesterase gene. This is the first report of carbamate resistance out of southern Africa as no carbamate resistance was observed in Uganda (East Africa) [7] or in Ghana [10]. The underlying mechanisms of this resistance remained to be established but could probably be metabolic as no evidence of an altered acetylcholinesterase was observed and no G119S mutation is present in this population. The bendiocarb resistance recorded in this study raises a special concern for National Malaria Control programs which, because of high resistance to pyrethroids and DDT, are currently introducing bendiocarb based IRS for malaria vector control in West African countries.

The full susceptibility observed against malathion is similar to all the tests carried out so far on *An. funestus* populations across Africa indicating that this insecticide could be used as an alternative to pyrethroids, DDT or carbamates for IRS against this species. Only a relatively moderate resistance against dieldrin was observed for females while males were fully susceptible. This result is in accordance with recent genotyping results of the *RDL^r^* allele in this population with only a frequency of 16% found in Pahou contrary to 82% in population in Cameroon where a higher level of dieldrin resistance is reported [9].

The underlying mechanism of the multiple resistance pattern observed in this populations was explored through various means. The synergist assay with PBO, an inhibitor of Cytochrome P450 monooxygenases, indicated that this enzyme family plays no role or little in the high DDT resistance observed in Pahou. Indeed, the mortality rate to DDT does not significantly vary either when mosquitoes are pre-exposed to PBO or not. The *kdr* L1014F or L1014S change commonly associated with pyrethroid/DDT resistance in other mosquitoes such as *An. gambiae* was not detected in this population of *An. funestus* as it has also been the case for all populations of this species analysed so far. The absence of this mutation in the Pahou *An. funestus* population does not support the presence of a cross-resistance mechanism between DDT and pyrethroids contrary to *An. gambiae*. This is further supported by the high difference in the level of resistance to DDT and pyrethroids in Pahou.

However, despite the absence of the L1014F/S mutation, a correlation was observed between the haplotypes of two fragments of the VGSC gene and the permethrin resistance phenotype. For each of the fragments spanning exon 20 and exons 27 to 31 respectively, a clade for resistant mosquitoes and another for susceptible were consistently observed as similarly observed for an *An. funestus* population in Uganda [7]. This is an indication that although no *kdr* mutation has been found in the exons 20 and 27 to 31, it is likely that one or more mutations located in other exons of the Voltage-gated sodium channel (VGSC) gene may be conferring the knockdown resistance in the *An. funestus* population of Pahou. It cannot also be excluded that part of the high DDT resistance might be associated to such mutations. Therefore, more exons should be sequenced particularly those where other mutations have previously been associated with knockdown resistance in other insects such as the exons 19 and 21 [34].

Both biochemical assays and qPCR results suggested that metabolic resistance is involved in the resistance to DDT, pyrethroids and carbamates. The elevated level of cytochrome monooxygenases from the biochemical assays was confirmed by the significant over-expression of two duplicated P450s, *CYP6P9a* and *CYP6P9b*, previously found to confer pyrethroid resistance in either laboratory or field populations [6], [11], [12]. However, not enough mosquitoes were available to carry out the PBO synergist assay with pyrethroids and carbamate to further confirm P450s involvement in these resistance patterns. However the level of expression of these two genes is significantly lower than that observed in southern Africa. This difference can also explain the lower resistance level observed in Pahou compared to the high resistance level in Mozambique. Indeed, compared to the FANG susceptible strain, *CYP6P9b* was over-expressed 15-fold in the *An. funestus* population from Mozambique while this is down to only 7-fold for Pahou. *CYP6P9a* was over-expressed 12-fold in Mozambique compared to only 4.7-fold in Benin. It remains to be confirmed whether these two genes are the main candidate in pyrethroid resistance in Benin as seen in southern Africa and to answer this question, a more comprehensive transcriptome profiling using tools such as microarray will be needed. However, *CYP6P3*, an ortholog of *CYP6P9* in *An. gambiae,* was also found to be associated with pyrethroids resistance in South Benin [3] further supporting a potential involvement for both copies of *CYP6P9* in pyrethroids resistance in *An. funestus* from Benin. Analysis of another P450 gene *CYP6Z1* did not indicate a significant involvement of this gene in the resistance pattern in Pahou contrary to observations made in some *An. gambiae* populations where this gene has been associated with both pyrethroid and DDT resistance [16], [35].

The significantly high level of GSTs observed using the biochemical assay was also supported by a significant over-expression of the *GSTe2* gene in the Pahou population compared to the FANG susceptible strain. Noticeably, this level was significantly higher in DDT resistant mosquitoes compared to the permethrin resistant or in mosquitoes not exposed to insecticides indicating that *GSTe2* is likely to play a significant role in DDT resistance in the Pahou populations. This will not be surprising as this gene has been shown to confer DDT resistance in other mosquito species such as *An. gambiae* and *Ae. aegypti* [16], [28], [36].

### Conclusion

This study has provided the first assessment of the susceptibility to the main insecticides used in public health of an *An. funestus* population from Benin and also explored the possible mechanisms responsible for the multiple resistance observed. The multiple resistance profile observed in the Pahou population highlights the need for further studies to assess the extent and the geographical distribution of these resistances in *An. funestus* populations in Benin and West Africa as well as a more comprehensive analysis of the resistance mechanisms involved. This will improve the implementation and management of future control programs against this important malaria vector in Benin and in Africa in general.

## References

[1] Corbel V, N'Guessan R, Brengues C, Chandre F, Djogbenou L (2007). Multiple insecticide resistance mechanisms in *Anopheles gambiae* and *Culex quinquefasciatus* from Benin, West Africa..

[2] Djogbenou L, Pasteur N, Akogbeto M, Weill M, Chandre F (2010). Insecticide resistance in the *Anopheles gambiae* complex in Benin: a nationwide survey..

[3] Djouaka RF, Bakare AA, Coulibaly ON, Akogbeto MC, Ranson H (2008). Expression of the cytochrome P450s, CYP6P3 and CYP6M2 are significantly elevated in multiple pyrethroid resistant populations of *Anopheles gambiae s.s.* from Southern Benin and Nigeria.. BMC Genomics.

[4] Brooke BD, Kloke G, Hunt RH, Koekemoer LL, Temu EA (2001). Bioassay and biochemical analyses of insecticide resistance in southern African *Anopheles funestus* (Diptera: Culicidae).. Bull Entomol Res.

[5] Casimiro S, Coleman M, Mohloai P, Hemingway J, Sharp B (2006). Insecticide resistance in *Anopheles funestus* (Diptera: Culicidae) from Mozambique.. J Med Entomol.

[6] Cuamba N, Morgan JC, Irving H, Steven A, Wondji CS (2010). High level of pyrethroid resistance in an *Anopheles funestus* population of the Chokwe District in Mozambique.. PLoS One.

[7] Morgan JC, Irving H, Okedi LM, Steven A, Wondji CS (2010). Pyrethroid resistance in an *Anopheles funestus* population from Uganda.. PLoS One.

[8] Hargreaves K, Koekemoer LL, Brooke BD, Hunt RH, Mthembu J (2000). *Anopheles funestus* resistant to pyrethroid insecticides in South Africa.. Med Vet Entomol.

[9] Wondji CS, Dabire RK, Tukur Z, Irving H, Djouaka R (2011). Identification and distribution of a GABA receptor mutation conferring dieldrin resistance in the malaria vector *Anopheles funestus* in Africa.. Insect Biochem Mol Biol.

[10] Okoye PN, Brooke BD, Koekemoer LL, Hunt RH, Coetzee M (2008). Characterisation of DDT, pyrethroid and carbamate resistance in *Anopheles funestus* from Obuasi, Ghana.. Trans R Soc Trop Med Hyg.

[11] Wondji CS, Irving H, Morgan J, Lobo NF, Collins FH (2009). Two duplicated P450 genes are associated with pyrethroid resistance in *Anopheles funestus*, a major malaria vector.. Genome Res.

[12] Amenya DA, Naguran R, Lo TC, Ranson H, Spillings BL (2008). Over expression of a cytochrome P450 (CYP6P9) in a major African malaria vector, *Anopheles Funestus*, resistant to pyrethroids.. Insect Mol Biol.

[13] Gillies MT, Coetzee M (1987). A supplement to the Anophelinae of Africa south of the Sahara (Afrotropical region)..

[14] Koekemoer LL, Kamau L, Hunt RH, Coetzee M (2002). A cocktail polymerase chain reaction assay to identify members of the *Anopheles funestus* (Diptera: Culicidae) group.. Am J Trop Med Hyg.

[15] WHO, WHO/CDS/CPC/MAL/98.12 D (1998). Test procedures for insecticide resistance montoring in malaria vectors, bio-efficacy and persistence of insecticides on treated surfaces..

[16] David JP, Strode C, Vontas J, Nikou D, Vaughan A (2005). The *Anopheles gambiae* detoxification chip: a highly specific microarray to study metabolic-based insecticide resistance in malaria vectors.. Proc Natl Acad Sci U S A.

[17] Penilla RP, Rodriguez AD, Hemingway J, Torres JL, Arredondo-Jimenez JI (1998). Resistance management strategies in malaria vector mosquito control. Baseline data for a large-scale field trial against *Anopheles albimanus* in Mexico.. Med Vet Entomol.

[18] Vulule JM, Beach RF, Atieli FK, Roberts JM, Mount DL (1994). Reduced susceptibility of *Anopheles gambiae* to permethrin associated with the use of permethrin-impregnated bednets and curtains in Kenya.. Med Vet Entomol.

[19] Martinez-Torres D, Chandre F, Williamson MS, Darriet F, Berge JB (1998). Molecular characterization of pyrethroid knockdown resistance (kdr) in the major malaria vector *Anopheles gambiae s.s.*. Insect Mol Biol.

[20] Ranson H, Jensen B, Vulule JM, Wang X, Hemingway J (2000). Identification of a point mutation in the voltage-gated sodium channel gene of Kenyan *Anopheles gambiae* associated with resistance to DDT and pyrethroids.. Insect Mol Biol.

[21] Harris AF, Rajatileka S, Ranson H (2010). Pyrethroid resistance in *Aedes aegypti* from Grand Cayman.. Am J Trop Med Hyg.

[22] Collins FH, Mendez MA, Rasmussen MO, Mehaffey PC, Besansky NJ (1987). A ribosomal RNA gene probe differentiates member species of the *Anopheles gambiae* complex.. Am J Trop Med Hyg.

[23] Thompson JD, Higgins DG, Gibson TJ (1994). CLUSTAL W: improving the sensitivity of progressive multiple sequence alignment through sequence weighting, position-specific gap penalties and weight matrix choice.. Nucleic Acids Res.

[24] Librado P, Rozas J (2009). DnaSP v5: a software for comprehensive analysis of DNA polymorphism data.. Bioinformatics.

[25] Tamura K, Dudley J, Nei M, Kumar S (2007). MEGA4: Molecular Evolutionary Genetics Analysis (MEGA) software version 4.0.. Mol Biol Evol.

[26] Wondji CS, Priyanka De Silva WA, Hemingway J, Ranson H, Parakrama Karunaratne SH (2008). Characterization of knockdown resistance in DDT- and pyrethroid-resistant *Culex quinquefasciatus* populations from Sri Lanka.. Trop Med Int Health.

[27] Wondji CS, Morgan JC, Coetzee M, Hunt R, Steen K (2007). Mapping a Quantitative Trait Locus conferring pyrethroid resistance in the African malaria vector *Anopheles funestus*.. BMC Genomics.

[28] Lumjuan N, Rajatileka S, Changsom D, Wicheer J, Leelapat P (2011). The role of the Aedes aegypti Epsilon glutathione transferases in conferring resistance to DDT and pyrethroid insecticides.. Insect Biochem Mol Biol.

[29] Gregory R, Darby AC, Irving H, Coulibaly MB, Hughes M (2011). A de novo expression profiling of *Anopheles funestus*, malaria vector in Africa, using 454 pyrosequencing.. PLoS One.

[30] Hunt RH, Brooke BD, Pillay C, Koekemoer LL, Coetzee M (2005). Laboratory selection for and characteristics of pyrethroid resistance in the malaria vector *Anopheles funestus*.. Med Vet Entomol.

[31] Koekemoer LL, Kamau L, Garros C, Manguin S, Hunt RH (2006). Impact of the Rift Valley on restriction fragment length polymorphism typing of the major African malaria vector *Anopheles funestus* (Diptera: Culicidae).. J Med Entomol.

[32] Michel AP, Ingrasci MJ, Schemerhorn BJ, Kern M, Le Goff G (2005). Rangewide population genetic structure of the African malaria vector *Anopheles funestus*.. Mol Ecol.

[33] Dabire KR, Baldet T, Diabate A, Dia I, Costantini C (2007). *Anopheles funestus* (Diptera: Culicidae) in a humid savannah area of western Burkina Faso: bionomics, insecticide resistance status, and role in malaria transmission.. J Med Entomol.

[34] Saavedra-Rodriguez K, Urdaneta-Marquez L, Rajatileka S, Moulton M, Flores AE (2007). A mutation in the voltage-gated sodium channel gene associated with pyrethroid resistance in Latin American *Aedes aegypti*.. Insect Mol Biol.

[35] Chiu TL, Wen Z, Rupasinghe SG, Schuler MA (2008). Comparative molecular modeling of *Anopheles gambiae* CYP6Z1, a mosquito P450 capable of metabolizing DDT.. Proc Natl Acad Sci U S A.

[36] Strode C, Wondji CS, David JP, Hawkes NJ, Lumjuan N (2008). Genomic analysis of detoxification genes in the mosquito *Aedes aegypti*.. Insect Biochem Mol Biol.

